# Reconstruction of Foot and Ankle Defects Using Free Lateral Arm Flap: A Retrospective Review of Its Versatile Application

**DOI:** 10.1155/2021/4128827

**Published:** 2021-10-31

**Authors:** Jong-Ho Kim, Taekeun Yoon, Joseph Kyu-hyung Park, Seokchan Eun

**Affiliations:** Department of Plastic and Reconstructive Surgery, Seoul National University College of Medicine, Seoul National University Bundang Hospital, Republic of Korea

## Abstract

**Background:**

Successful reconstruction of the feet and ankles remains challenging due to limited quantities of soft tissue and laxity. The free lateral arm flap (LAF) is an alternative to conventional flaps and has been widely used due to advancements in its flap characteristics. This study is aimed at utilizing the advantages of this flap to validate its increased applications for foot and ankle defects.

**Methods:**

Twenty patients with various LAF types between May 2011 and May 2020 were enrolled. Clinical data was retrospectively collected, and defect sites were classified according to the subunit principle. We utilized various LAF types, such as LAFs with sensate, extended, osteomyocutaneous, or myocutaneous flaps, as necessary. A two-point discrimination test was performed, and results were statistically compared between flaps.

**Results:**

Among the diverse etiologies of skin defects, chronic inflammation was the most common cause of defects. Various LAF types, including LAFs with fasciocutaneous, extended fasciocutaneous, musculocutaneous, and osteomyocutaneous flaps, were used. The versatility of free LAF helped successfully cover various defects in all cases. Results of the two-point discrimination test were statistically significant between groups.

**Conclusions:**

Free LAF is a unique soft tissue free flap that is more versatile than other flaps, allowing flaps to be continuously modified and applied to various foot and ankle defects under different clinical conditions.

## 1. Introduction

Managing defects in the distal lower extremities is challenging, and covering this region usually requires a local or free flap. Reconstructing soft tissue defects, especially around the foot and ankle, is difficult for reconstructive surgeons due to the relatively poor circulation, structural and histological characteristics, and lack of locally available tissues [1]. Thin skin or weight bearing should always be considered for reconstructing feet and ankles [2]. Hollenbeck et al. suggested the application of the subunit principle for the treatment of foot and ankle wounds [3]. In their study, the ankle is divided into two subunits: lateral and medial malleoli, the dorsal foot is divided into 3 subunits: dorsal hind foot, dorsal midfoot, and dorsal forefoot, and the plantar foot is divided into 3 subunits: plantar heel, plantar midfoot, and plantar forefoot. They noted that reconstructing these areas was difficult due to the needs of each region. Various flaps have been introduced to overcome the corresponding challenges, and the free lateral arm flap (LAF) has been widely considered as the treatment of choice due to its various advantages and applicability in numerous areas [4]. There has been few discussion about the usefulness of LAF compared to other flaps in the reconstruction of foot and ankle. These advantages include the ease of elevation as a chimeric flap or sensate flap. It is applicable to various defect sites and can be applied through several methods. For instance, it can be used as a fasciocutaneous flap, a myocutaneous flap with a portion of the triceps muscle, and an osteocutaneous flap with a block of the distal humerus [5]. LAF has been reported to distally extend beyond the elbow and provide a thin and pliable flap [6, 7]. Moreover, the advantages of this flap include relatively easy dissection, persistent vascular anatomy, and minimal donor site morbidity. We herein present our cases in which free LAF was used to reconstruct the foot and ankle to elucidate its versatile application.

## 2. Patients and Methods

Twenty patients with various foot and ankle defects treated with free LAF between May 2011 and May 2020 were enrolled in this study. This study was approved by the institutional review board of the Seoul National University Bundang Hospital. A retrospective chart review of clinical data was developed, analyzing patient characteristics, lesion location, etiologies, comorbidities, flap size, type, types of anastomosis, recipient vessels, applications of sensate flaps, and postoperative complications (Table 1). Defect sites were classified according to the subunit principle of Hollenbeck et al. [3]. In cases in which sensate flaps are used for reconstruction, the two-point discrimination test was performed to evaluate sensory function on the central portion of the flap. Statistical analysis was performed using R-Studio version 0.98.953. Based on the data distribution tested with the Shapiro–Wilk test, Student's *t*-test was performed.

If the size of the defect was large, an extended flap covering the distal portion of the elbow level was used. A chimeric myocutaneous or osteomyocutaneous flap was used to control chronic infection or fill the dead space of a lesion, if necessary. The posterior or lateral brachial cutaneous nerve was separated during flap elevation and subsequently coapted to the cutaneous nerve of recipient sites if a sensate flap was planned to be used.

## 3. Results

Our patient cohort consisted of eight (40%) men and 12 (60%) women, with a mean age of 66.8 (range, 48–89) years. The various defect sites observed in this cohort are summarized in Table 1. Among the diverse etiologies of skin defects, different kinds of chronic inflammation, including chronic osteomyelitis and ulcers (*n* = 9), followed by malignant melanoma (*n* = 4), diabetic foot (*n* = 3), trauma (*n* = 3), and burn injury (*n* = 1), were the most common cause of defects. Regarding the elevation type of free LAF, fasciocutaneous free LAF was performed in 16 cases, extended fasciocutaneous free LAF in two cases, myocutaneous free LAF in one case, and osteomyocutaneous free LAF in one case. Sensate free LAF was usually applied in patients who had plantar defects. In the two-point sensory discrimination test, the distinguishable distance in sensate flaps was 19.1 ± 4.9 mm, whereas that in nonsensate flaps was 28.9 ± 4.2 mm. This between-group difference was statistically significant (*P* < 0.05, *t*-test using R-studio). The mean follow-up period was 18.2 (range, 9–42) months.

LAF dimensions ranged from 12 to 108 cm^2^ (mean, 48.7). The average pedicle length was 6.4 cm, with a maximal length of 9 cm. Veins requiring microanastomosis were anastomosed using the end-to-end technique. Moreover, 12 and 8 cases requiring arterial anastomosis were anastomosed using the end-to-side and end-to-end techniques, respectively. There were no cases of total flap loss. As for complications, two patients demonstrated flap congestions on postoperative monitoring that were successfully treated using vein reanastomosis, and three other patients showed partial necroses that were treated using flap readvancement or split thickness skin grafting. All patients who demonstrated flap congestion and two of the three patients who showed partial necrosis had a history of diabetes mellitus. No late infective complications occurred. Flap debulking surgery was performed in one case. All donor sites were primarily closed, and there were no major donor site-related complications. All patients showed good postoperative donor arm function and no signs of radial nerve injuries.

### 3.1. Case 1 (Patient Number 19)

**Figure 1:**
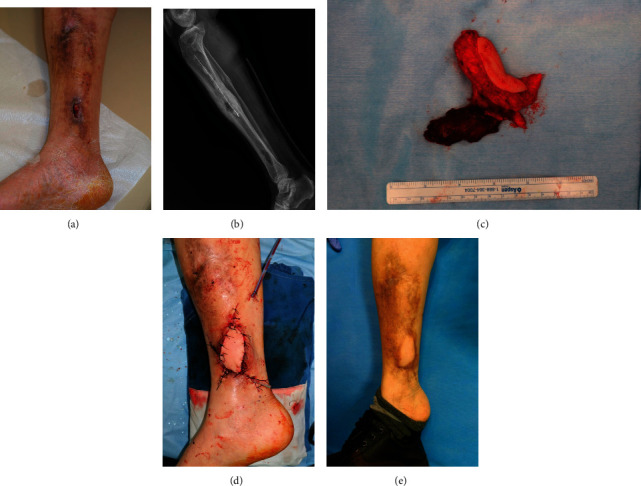
(a) A 58-year-old male with persistent wound problem on right distal leg. (b) Preoperative lower leg X-ray showed osteolytic lesion on tibia. (c) Harvested lateral arm free flap. (d) Immediate postoperative image. (e) Postoperative 10 months.

A 58-year-old male patient was referred to our outpatient clinic due to persistent wound problems on his right distal leg (Figure 1(a)). He had undergone open reduction and internal fixation due to right distal tibiofibular fracture caused by a traffic accident. Two months postoperatively, his implant was removed and antibiotic beads were inserted as osteomyelitis was suspected. Despite repeated debridement and antibiotic treatment, his skin defect did not show any improvement, and the patient was referred to our department. On lower leg radiography, osteolytic lesions were observed at the right tibia shaft and medial malleolus area (Figure 1(b)). The amount of soft tissue in the medial side of the distal tibia was insufficient for a local flap, and inflammation progression was difficult to control. Accordingly, reconstruction with a 5 × 3 cm chimeric myocutaneous LAF with the lateral head of the triceps was planned (Figure 1(c)). Following lesion debridement and flap elevation, end-to-side anastomosis was microscopically performed between the posterior tibial vessels using the posterior radial collateral vessels. The muscular portion of the flap was inserted into the deep tissue defect for infection control (Figure 1(d)). No complications were observed 10 months postoperatively (Figure 1(e)).

### 3.2. Case 2 (Patient Number 17)

**Figure 2:**
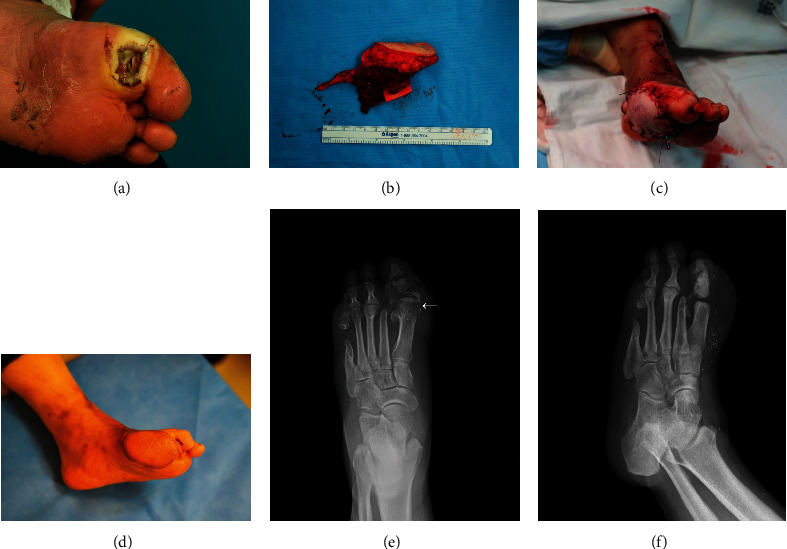
(a) A 50-year-old woman with an recurrent diabetic foot ulcer involving the big toe. (b) Harvested free lateral arm osteomyocutaneous flap. (c) Immediate postoperative image. (d) Postoperative 13 months. (e) Preoperative X-ray image shows osteomyelitis lesion from the proximal phalanx of the left first toe (white arrows). (f) Postoperative X-ray image shows successful bone replacement by filling the first proximal phalanx with the bony portion of osteomyocutaneous flap.

A 50-year-old woman with an infection on her left big toe that lasted for 3 months was admitted to our department. She had a history of uncontrolled diabetes affecting her left foot had her second and fifth toes amputated at our orthopedic department 6 years ago (Figure 2(a)). Diagnostic left lower extremity arteriography was performed to determine the appropriate surgical plan. At the infra-ankle level, metatarsal artery branching from the posterior tibial artery was observed in the forefoot portion, and the dorsalis pedis artery was absent. The metatarsal artery of the first toe was not visible, and supply vessels comprised multiple fine collaterals. Accordingly, the infected unhealthy bone was planned to be debrided along with the insertion of a free lateral osteomyocutaneous flap (Figure 2(b)). An anastomosis connecting the medial plantar and posterior radial collateral arteries was performed using the end-to-side method. Two concomitant flap veins were each anastomosed to the medial plantar vein and surrounding cutaneous vein. The osteomuscular portion of the flap was inserted and fixed to the first metatarsal defect (Figure 2(c)). In postoperative follow-up, the great toe was excised due to ischemic change and instability after intraoperative debridement. No complications, such as flap infection, ulceration, or flap loss, were observed in during a 13-month follow-up session (Figures 2(d)–2(f)).

### 3.3. Case 3 (Patient Number 9)

**Figure 3:**
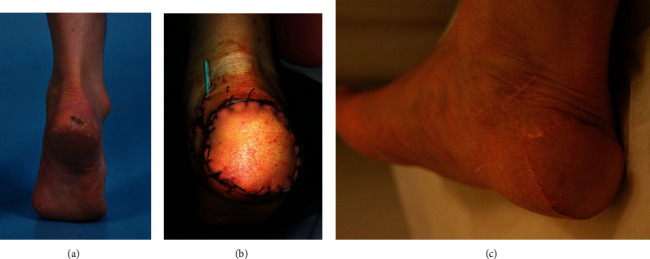
(a) A 78-year-old man with malignant melanoma on the right heel. (b) Immediate postoperative image. (c) Postoperative 12 months.

A 78-year-old man presented with a 2 × 1 cm lesion on his right heel that was confirmed to be a malignant melanoma on punch biopsy (Figure 3(a)). No metastatic lesions were observed on radiological examination. Wide tumor excision was planned, with resection margins of 2 cm, as clearly observed on intraoperative frozen observed. A 5 × 4 cm LAF flap was elevated, and vessels were anastomosed to the posterior tibial artery and its vena comitans using the end-to-side and end-to-end methods, respectively (Figure 3(b)). There were no immediate postoperative complications. Twelve months postoperatively, the patient showed no sign of recurrence and was satisfied with both functional and esthetic outcomes (Figure 3(c)).

## 4. Discussion

### 4.1. Basic Principles of Foot and Ankle Reconstruction

The basic principles of lower extremity reconstruction include replacement with similar tissues, minimization of donor site problems, and preservation of main vessels [8]. In cases in which there are many lower extremity wounds, the comprehensive assessment of circulation, tissue deficiency, and bony or muscular injuries is essential to ensure optimal outcomes [9]. Skin grafts and local flaps may be sufficient for the proper reconstruction of lower extremities in some cases. For reconstruction of the heel, the medial plantar flap is a good option. However, in some cases, it cannot adequately cover the back of the heel because the pedicle length is short (case 3) and an osteomyocutaneous flap with sufficient blood supply is required to control osteomyelitis and replace the injured bone (case 2); a free flap should be used in these cases [10]. Because of the diverse characteristics of the foot and ankle area, various factors, including functional and esthetic aspects, should be considered [11]. The weight-bearing region is a unique part of our body, and its characteristics are completely different from that of the dorsal foot; flaps should be able to endure bodyweight and shearing force. However, thinner flaps that do not interfere with ambulation or the wearing of shoes in the dorsal foot or ankle region should be considered.

### 4.2. Characteristics and Advantages of LAF

Compared to an anterolateral thigh (ALT) flap, which is widely used for lower extremity reconstruction, free LAF has a thinner flap, cosmetic excellence, and a lower risk of debulking procedure-induced partial loss. Moreover, patients who underwent LAF surgery can quickly recover and return to normally performing activities of daily life [5]. LAF is usually thicker than the radial forearm flap, which is commonly used for dorsal foot reconstruction. In select cases, LAF can be designed to have regular or thin thickness using a distal perforator-based design, if necessary [12], providing an effective reconstructive design when both thick and thin parts are simultaneously required. This is usually common especially in defects including the toe.

LAF is considered to be relatively short compared to the radial forearm or ALT flaps. However, the disadvantages of a short pedicle can be solved by detaching the proximal head of the triceps muscle and dissecting up to the level of the deep brachial artery in the spiral groove [12, 13]. If a pedicle length of >9 cm was required, interpositional vessel grafting is not performed. All flaps can be successfully utilized within this pedicle range. Moreover, in this study, various LAF types, including fasciocutaneous, musculocutaneous, and osteocutaneous LAFs, were used, broadening LAF applicability.

### 4.3. Flap Size and Application of Chimeric LAF

In our study, flap sizes ranged from 12 to 108 cm^2^. Conventionally, the size of fasciocutaneous flaps ranges from 9 to 20 cm in length and 3–8 cm in width, and distally extended LAFs can be used to cover wide defects [6]. In addition to the advantages of extended flaps, interestingly, LAF has a benefit in that it can be harvested as a small flap. The relatively short distance between fascia and skin, together with a well-visible perforator, enables the easy harvest of small flaps. Although all types of small flaps can be elevated, it is easier to elevate small LAFs as chimeric flaps than as other flaps since the muscular or bony portions of defects are located close to the flap [14]. In cases of both osteofasciocutaneous flaps with distal humerus and musculofasciocutaneous flaps with triceps components, LAF has greater accessibility to different tissue components than ALT and superficial circumflex iliac artery perforator (SCIP), scapular, and parascapular flaps. SCIP flap has superior advantages in lower extremity reconstruction, namely, thin thickness, a hidden scar, and simultaneous elevation of the iliac bone. However, compared to LAF, it takes longer time and more effort to elevate the flap with the bone or muscle (sartorius muscle or iliac bone), and the nerve anatomy is not established as a sensate flap. As for dead spaces or regions with a high risk of repeated infection, it is often necessary to insert not only fatty tissue, but also more tolerable tissues, such as fascia or muscle. In cases 1 and 2, we successfully applied chimeric flaps and were accordingly able to control repeated infection and chronic ulceration. Thus, LAF should be remembered as a great option if durable tissue needs to be inserted into a relatively small defect.

### 4.4. Use of Free Sensate LAF

We advocate the necessity of thick flaps in plantar reconstruction; however, LAF shoulder be considered as an important option in weight-bearing region reconstruction due to the advantages of its sensate flaps. Among the free flaps commonly used for foot and ankle reconstruction, LAF is relatively easy to elevate as a sensate flap due to the persistent location of cutaneous nerves [15]. Some studies have reported that the sensations of plantar flaps ultimately returned, regardless of whether neurorrhaphy was performed [16, 17]. However, ulcerative or chronic inflammatory lesions can reduce the spontaneous sensory recovery rate unless a sensate flap is used [18]. Other studies have demonstrated that sensate flaps lend better long-term foot weight-bearing surface reconstruction outcomes than other flaps [19, 20]. In our experiences, sensory preservation is crucial to obtain long-term outcomes, especially in patients at risk of recurrent ulcers.

This study has several limitations. First, the sample size of this study was not enough for statistical analysis, and LAF was not compared with other local flaps. Second, this study was retrospective; bias may have occurred since this study only included patients we thought could be successfully treated with free LAF. Prospective studies with more patients to evaluate long-term sensate flap outcomes are warranted.

## 5. Conclusions

We herein demonstrated that free LAF is effective for various foot and ankle defects and justified the widespread use of this flap. Despite the small sample size of this study, we believe that this study provides further insights on the extended application of LAF, which can be used in various ways.

## Figures and Tables

**Table 1:** Patient demographics and operative data.

	Age/sex	Location	Etiology	Comorbities	Flap size (cm^2^) and type	Anastomosis (artery, vein)	Recipient vessels (A/V)	Sensate flap/2PD (mm)	Postoperative complications
1	78/M	Medial malleolus & dorsal hindfoot	Skin necrosis (cellulitis)	DM	12 × 4, FC	ES, EE	PTA/PTV	-/31	-
2	50/M	Lateral malleolus	Chronic ulcer	Smoke	4 × 3, FC	EE, EE	ATA/ATV, cutaneous vein	-/30	-
3	72/F	Lateral malleolus	Chronic osteomyelitis		10 × 6, FC	ES, EE	ATA/ATV	-/28	-
4	48/F	Plantar forefoot (1st toe)	Malignant melanoma		12 × 5, FC	EE, EE	MPA/MPV	+/14	Debulking
5	77/F	Plantar forefoot	Malignant melanoma	DM	6 × 4, FC	EE, EE	MPA/cutaneous vein	+/28	Partial necrosis
6	69/M	Plantar forefoot	Diabetic foot	HTN, DM	10 × 7, FC	ES, EE		-/32	-
7	89/F	Dorsal mid & forefoot	Burn	HTN, DM	18 × 6, Ext. FC	EE, EE	DPA/DPV	-/32	Marginal necrosis
8	78/F	Medial malleolus	Chronic osteomyelitis		20 × 5, Ext. FC	ES, EE	PTA/PTV	-/25	-
9	78/M	Plantar heel	Malignant melanoma		5 × 4, FC	ES, EE	PTA/PTV	+/16	-
10	49/M	Plantar heel	Chronic ulcer		8 × 6, FC	ES, EE	PTA/PTV	-/28	-
11	49/M	Dorsal mid & forefoot	Trauma		15 × 6, FC	EE, EE	DPA/DPV	+/23	-
12	70/M	Plantar midfoot	Malignant melanoma		9 × 7, FC	ES, EE	PTA/PTV	+/20	-
13	72/M	Medial malleolus	Chronic osteomyelitis	DM, HTN, KTPL	6 × 5, FC	ES, EE	PTA/PTV	-/25	Flap congestion & reanastomosis (vein)
14	65/M	Dorsal mid & forefoot	Degloving injury		11 × 6, FC	EE, EE	DPA/DPV	+/17	-
15	73/F	Dorsal midfoot	Chronic ulcer	Epilepsy	7 × 4, FC	ES, EE	ATA/ATV	-/20	Proximal margin necrosis
16	58/M	Plantar & dorsal forefoot	Chronic ulcer	DM	6 × 5, FC	EE, EE	1st dorsal metatarsal artery/cutaneous vein	-/33	Flap congestion & reanastomosis (vein)
17	50/F	Plantar forefoot	Diabetic foot	DM	6 × 5, OMC	ES, EE	MPA/MPV, cutaneous vein	-/35	-
18	73/F	Dorsal forefoot	Trauma		9 × 5, FC	EE, EE	DPA/DPV, cutaneous vein	-/25	-
19	58/M	Medial malleolus	Chronic osteomyelitis	DM, HTN	5 × 3, MC	ES, EE	PTA/PTV	-/32	-
20	70/M	Plantar heel	Diabetic foot	DM	9 × 3, FC	ES, EE	PTV/PTV	+/16	-

DM: diabetes mellitus; HTN: hypertension; KTPL: kidney transplantation; FC: fasciocutaenous flap; Ext.: extended; OMC: osteomyocutaneous flap; MC: musculocutaneous flap; ES: end to side; EE: end to end; PTA: posterior tibial artery; PTV: posterior tibial vein; ATA: anterior tibial artery; ATV: anterior tibial vein; MPA: medial plantar artery; MPV: medial plantar vein; DPA: dorsalis pedis artery; DPV: dorsalis pedis vein; 2PD: 2-point discrimination test.

## Data Availability

All datasets that the conclusion is based upon are referred in the manuscript text. The datasets analyzed during the current study are available from the corresponding author on reasonable request.

## Abbreviations

LAF: Lateral arm flap
ALT: Anterolateral thigh
SCIP: Superficial circumflex iliac artery perforator.
